# Prospective clinical study of sentinel node detection in bladder cancer using a hybrid tracer – Towards replacement of pelvic lymph node dissection in cases with sentinel node visualization on SPECT/CT?

**DOI:** 10.1007/s00259-025-07240-z

**Published:** 2025-04-04

**Authors:** E. J. van Gennep, G. Pisano, G. H. KleinJan, D. D. D. Rietbergen, K. Hendricksen, L. S. Mertens, M. W. vd Kamp, E. M. K. Wit, M. L. van Montfoort, M. Donswijk, B. W. G. van Rhijn, F. W. B. van Leeuwen, H. G. van der Poel

**Affiliations:** 1 Present Address: Department of Urology, Leiden University Medical Center, Leiden, The Netherlands; 2 Interventional Molecular Imaging Laboratory, Department of Radiology, Leiden University Medical Center, Leiden, The Netherlands; 3 Department of Nuclear Medicine, Leiden University Medical Center, Leiden, The Netherlands; 4 Department of Urology, Netherlands Cancer Institute-Antoni Van Leeuwenhoek Hospital, Amsterdam, The Netherlands; 5 Department of Pathology, Netherlands Cancer Institute-Antoni Van Leeuwenhoek Hospital, Amsterdam, The Netherlands; 6 Department of Nuclear Medicine, Netherlands Cancer Institute-Antoni Van Leeuwenhoek Hospital, Amsterdam, The Netherlands; 7 Department of Urology, Amsterdam University Medical Center, Amsterdam, The Netherlands

**Keywords:** Bladder cancer, Radio-guided surgery, Fluorescence, Robotic surgery, Sentinel lymph node

## Abstract

**Purpose:**

Nodal staging in patients with muscle invasive bladder cancer (MIBC) or very high risk non-muscle invasive bladder cancer (vhNMIBC) aids to predict survival. The sentinel node (SN) procedure holds the promise to identify the diagnostically relevant first tumor-draining nodes while limiting the complication rate associate with a pelvic lymph node dissection (PLND), still considered the gold standard of nodal staging. Following an initial technical feasibility study, we prospectively evaluated the clinical impact of using peri-tumoral injections with the hybrid tracer indocyanine green (ICG)-^99m^Tc-nanocolloid for SN procedures in bladder cancer*.*

**Methods:**

A total of 30 patients with vhNMIBC or MIBC, cN0M0 (with or without neo-adjuvant systemic therapy) scheduled for radical cystectomy with pelvic lymph node dissection (PLND) were enrolled in a prospective study. Patients received four to six transurethral peritumoral injections of ICG-^99m^Tc-nanocolloid into the bladder for SN tracing. Preoperative lymphoscintigraphy and Single Photon Emission Computed Tomography (SPECT)/CT was performed the day before surgery.

The intraoperative detection of SNs was supported by both fluorescence (utilizing a hand-held camera or fluorescence laparoscope) and radio-guidance (using hand-held, laparoscopic or DROP-IN probe tracing). Resected specimens, comprising both SNs and PLND tissue, were analyzed by the pathologist. Complications related to the tracer injection were documented and cancer-specific survival (CSS) and overall survival (OS) were studied using Kaplan–Meier survival curves.

**Results:**

SPECT/CT imaging revealed 31 SNs in 19 patients with non-visualization of SN in 11 patients (36.7%). During surgery, 4 additional SNs were identified based on fluorescent signals in 3 patients. In 1 patient who underwent open cystectomy, ex vivo evaluation of the PLND-specimen revealed an additional radioactive SN.

The PLND yielded 592 lymph nodes (LNs; median 17 LNs/patient). In 5 out of 35 SNs (14.2%; no additional tumor positive LN in complementary PLND) and 3 out of 592 LNs (0.5%; 2 patients with non-visualization of SN) were identified as tumor-positive upon pathological evaluation.

At mean follow up of 82 months (SD ± 7.1 months) 17% of patients died of disease. The 2 patients with non-visualization of SN and nodal metastases (0%) did worse than SN positive patients (75%). Of the 24 patients classified as pN0 8% died.

**Conclusions:**

Preoperative SN-visualization on SPECT/CT was achieved in the majority of patients. Patients with SN non-visualization had an increased risk of nodal metastases and poorer outcome. In patients with metastases in the SN no additional nodal metastases were found in the complementary PLND.

## Introduction

Bladder cancer (BC) ranks as the seventh most commonly diagnosed malignancy in the male population worldwide [1]. The local therapeutic strategies include radical cystectomy, and (chemo) radiation.

In BC, the identification of lymphatic spread serves as an independent prognostic factor influencing (nodal) recurrence and survival [2, 3]. Unfortunately, pelvic lymph node dissection (PLND) is associated with a spectrum of comorbidities, including vascular, ureteral, and nerve injuries, as well as complications such as lymphocele and thromboembolic events. Recently Lerner et al. showed no improvement in survival in patients treated with an extended PLND compared to patients treated with a standard PLND [4]. Consequently, it is imperative to minimize surgical morbidity by adopting a more targeted approach to nodal resections. Ideally, the strategy for nodal-resections focusses on minimizing the procedural invasiveness, meaning reduction of the extensiveness of the resection template and as such surgical and anesthesia time. Thus looking for strategies that identify the nodes that matter the most. Sentinel node (SN) procedures have been successfully introduced to guide nodal sampling in urological malignancies like penile and prostate cancer [5–8]. Here the SN is defined as the first lymph node (LN) receiving lymphatic tumor-drainage, making it the LN that preceeds systemic spread [9]. Sentinel node for BC patients was initially described by Sherif et al. [10]. Several studies using radio- [11, 12] or fluorescence- guidance [13, 14], and one with combined radio- and fluorescence-guidance (hybrid) [15] followed.

When radiocolloids are used to target the SNs, preoperative imaging can provide a surgical roadmap. Unfortunately, the lymphangiographic drainage profiles of “free” indocyanine green (ICG) differs from that of the radiocolloids, thus leading to potential discordances in findings [15, 16, 18]. Conversely, the hybrid tracer ICG-^99m^Tc-nanocolloid helps to assure pre- and intra-operative concordance by chemically integrating radio and fluorescence guidance [7, 17–19]. A strategy that has proven valuable in open, laparoscopic and robotic settings and thus aligns with the growing use of the robot in bladder surgery [13].

The primary objective of this prospective study is to evaluate the accuracy of the hybrid SN-procedure in patients with bladder cancer with a complementary PLND. Secondary objectives include assessment of the safety of the peri-tumoral hybrid tracer injections and to perform a survival analysis comparing pN0 versus pN + patients based on their SN-status.

## Methods

### Patients

This prospective trial was registered at the Netherlands Trial Register as NL48901.031.14 and was executed at the Netherlands Cancer Institute–Antoni van Leeuwenhoek Hospital after approval of the Medical Ethical Review Board of the institution (M14HSN). A subset of the included patients was also described in a previous study confirming feasibility [19].

Patients with histologically confirmed muscle invasive bladder cancer (MIBC) or very high risk non-muscle invasive bladder cancer (vhNMIBC) (cN0M0) were scheduled for SN-biopsy before radical cystectomy combined with PLND. Patients underwent transurethral tumor resection and were preoperatively staged using CT–intravenous urogram and ^18^F-FDG PET-CT, all staged clinically negative (cN0). All patients were prospectively included after informed consent. Neo-adjuvant chemotherapy (NAC) and neo-adjuvant immunotherapy (in case of clinical study) were allowed. If patients met the criteria for NAC (determined at multidisciplinary tumor-board meetings) they were treated as such and recorded for this study. The main exclusion criteria were suspected nodal disease on imaging and prior pelvic radiation and/or surgery.

### Sentinel node imaging procedure

On the day before surgery, a volume of 2 mL containing a mean dose of 206.5 MBq (IQR 197–221; 5.6 mCi) ICG-^99m^Tc-nanocolloid (GE-Healthcare, Leiderdorp, the Netherlands) was injected. Four to six transurethral injections were performed into the detrusor muscle of the bladder, around the tumor or transurethral resection scar, under flexible cystoscopic guidance using an endoscopic needle (Injetak; Laborie). This needle has a maximum 5-mm tip length. Static lymphoscintigraphy (15 min and 2 h post injection) as well as SPECT/CT (2 h post injection) were used to map the lymphatic drainage. A full description of the SPECT/CT scanning protocol was described by Rietbergen et al. [19]. Nodes were considered to be SNs when visualized as intense focal uptake on the early, delayed images and/or SPECT/CT, with increasing uptake in time or identified during surgery based on their fluorescent and/or radioactive signal. The number of SNs identified was recorded. Pre-operative detection rate was defined as the number of patients in whom at least one SN was identified at pre-operative imaging divided by the total number of injected patients.

### Surgical procedures

Patient underwent cystectomy in combination with PLND, either with an open procedure via laparotomy, or with a robotic approach laparoscopically. Patients scheduled for robot-assisted cystectomy were operated with the Da Vinci Xi system. Patients were counseled for cystectomy and neobladder reconstruction or cystectomy and ileal-conduit (Bricker). The number and location of surgically resected SNs was recorded.

The PLND template consisted of the LNs located medially from the ureter, extending along the external iliac artery, internal iliac artery, and within the obturator fossa.

### Fluorescence cameras

In open procedures, a handheld fluorescence camera (FIS-00, Hamamatsu, Japan) was used for fluorescence imaging. For the robot-assisted surgical procedure, the *da Vinci*® Xi Firefly fluorescence laparoscope (Intuitive Surgical Inc., Sunnyvale, CA, USA) was used.

### Gamma probes

A hand-held gamma probe (Eurorad S.A., Eckbolsheim, France) was used in open surgery and during ex vivo confirmatory studies. During robotic surgery a laparoscopic (Eurorad S.A., Eckbolsheim, France) or a tethered DROP-IN probe prototype (CE-marked Crystal DROP-IN, Crystal Photonics) were used. The DROP-IN probe was inserted into the abdominal cavity either through a trocar or through the Alexis® port (Alexis laparoscopic system, Applied Medical Corp., Rancho Santa Margarita, CA, USA) [20].

### Pathology

**Fig. 1:**
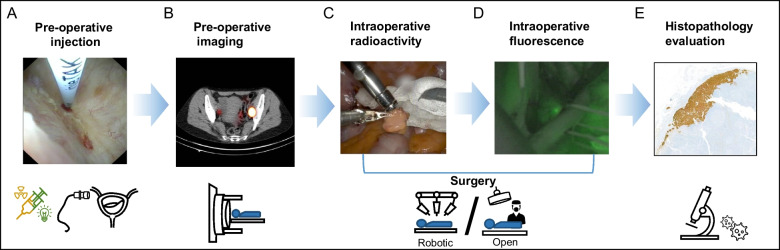
Graphic workflow of pre- and intra-operative imaging, surgical approach and histopathology. **A** Needle puncturing the bladder wall during flexible cystoscopy for injection of the hybrid tracer ICG-^99m^Tc-nanocolloid. **B** SPECT/CT showing bilateral SNs (**C**) Patients underwent either open or robotic surgery, aided by intra-operative radioactivity and (**D**) intra-operative fluorescence. **E** Histopathology evaluation of SN specimen. In brown Keratine AE 13/1 staining, containing metastasis. Abbreviations: ICG = indocyanin green, SPECT/CT = Single photon emission computed tomography – computed tomography, SN = sentinel node, PLND = pelvic lymph node dissection

SNs were assessed according to the SN-protocol of the department of pathology [17]. Additionally excised non-SNs were processed with the current standard procedures of the pathology department of the NKI-AvL. The total number of LNs removed and the number of LNs with metastatic cancer deposits were recorded. In Fig. 1, the graphic workflow of the SN-procedure is shown. Use of intraoperative gamma probe was based on the preference of the surgeon.

### Survival analysis

The Kaplan–Meier method was used to display overall survival and cancer specific survival. The log rank-test was used to assess significance, considering *P-*values of less than 0.05 as statistically significant. Version 29 of SPSS software (SPSS Inc.) was used.

## Results

### Patients

Between 2014–2023, 33 patients were asked for consent, finally 30 patients were included for the study and operated (Table 1). Of these, 23 were male (77%). The mean age of the patients was 64.3 years (day of radical cystectomy). Twenty-four (82%) patients were clinically staged as MIBC, cT2 and higher. Six patients (18.2%) with a very high-risk bladder cancer (eg. T1 + carcinoma in situ (CIS)) were included. Twelve patients (40%) received neo-adjuvant therapy, of whom 8 patients were treated with NAC and 4 with immunotherapy or a combination. As surgical approach, 22 patients underwent a robotic-assisted procedure (73%) and 8 patients underwent open procedure (27%).

**Table 1:** Patient characteristics

	Number of patients	Percentage
cT1	6	20%
cT2b	11	37%
cT3b	12	40%
cT4a	1	3%
NAC	8	27%
NA Immunotherapy	4	13%
pTis	4	13%
pT0	7	23%
pT1	3	10%
pT2a	1	3.0%
pT2b	7	23%
pT3a	4	13%
pT3b	4	13%
Concomitant CIS	7	23%
pN0	24	80%
pN1	5	17%
pN2	1	3%

Abbreviations: *c* clinical, *p* pathological, *CIS* carcinoma in situ, *NA* Neoadjuvant, *NAC* Neo adjuvant chemotherapy

Tumor (T) and Nodal (N)-stage

Eight patients had a cystectomy and neobladder reconstruction (27%) while 22 patients received cystectomy and ileal-conduit (73%). No side effects or complications were observed related to the cystoscopic injections of the hybrid tracer.

### Pre-operative SN identification (surgical roadmap)

SPECT/CT identified a total of 31 SN-related hotspots in 19 patients, yielding an average of 1.6 SNs per patient and a pre-operative detection rate of 63.3%. In 11 cases, no areas referable to SNs were visualized (36.7%). In the group of non-visualization of SN 5/11 (45%) patients received neo-adjuvant therapy.

**Fig. 2:**
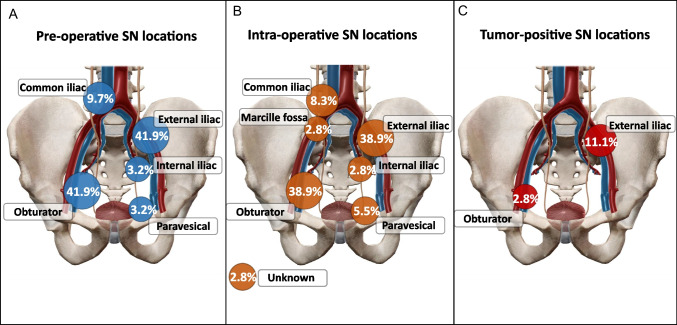
SN locations by anatomy. Graphical distribution of SNs locations: **A** At Pre-operative SPECT/CT imaging (13/31 SNs in Obturator area, 3/31 SNs in common iliac, 13/31 SNs in external iliac, 1/31 SNs in internal iliac, 1/31 SNs in paravesical area). **B** At intra-operative detection (14/35 SNs in Obturator area, 3/35 SNs in common iliac, 14/35 SNs in external iliac, 1/35 SN in internal iliac region, 2/35 SNs in paravesical area, 1/35 SNs in Marcille fossa, 1/35 unknown (detected ex vivo from PLND template)). **C** Of positive SNs at histopathology (4/35 SNs in external iliac, 1/35 SNs in obturator region found to harbor metastases). Abbreviations: SN = Sentinel node; SPECT/CT = Single photon emission computed tomography – computed tomography; PLND = pelvic lymph node disscetion

**Fig. 3:**
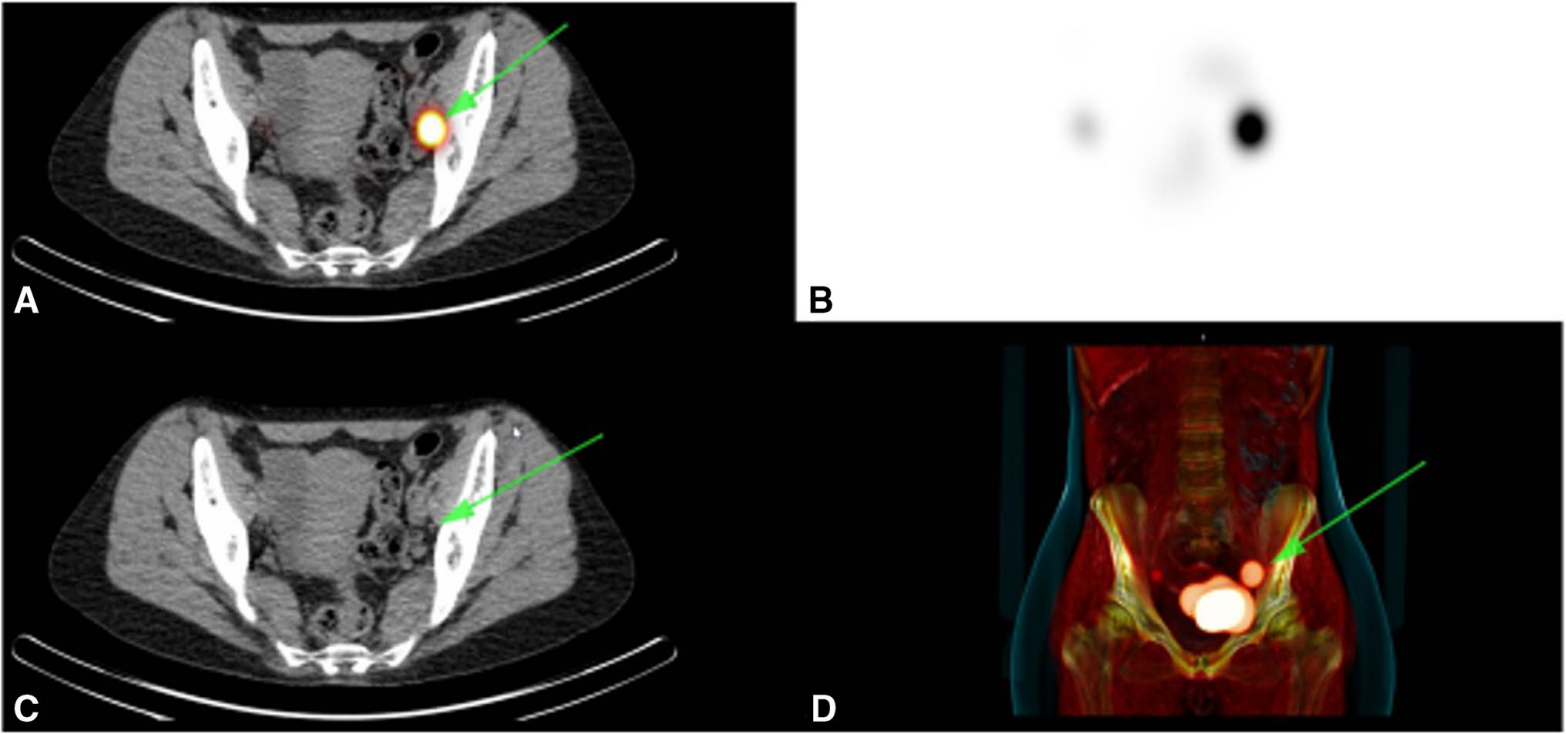
Preoperative SPECT/CT SN imaging. **A**: fusion of low-dose CT and SPECT image with localization of SN (green arrow). **B** SPECT imaging (**C**) Low dose CT with localization of SN (green arrow). **D** A 3D volume rendering of SPECT/CT image. In this patient, an intense SN was seen on the left obturator region marked with by the green arrow, on the right a faint SN was seen on SPECT/CT in the right obturator region, as better shown in the 3D volume rendering.. Abbreviations: CT = computed tomography; SPECT = Single photon emission computed tomography; SN = Sentinel node

SPECT/CT revealed SNs located in the obturator fossa (4 on the left and 9 on the right side), common Iliac trunks (1 on left and 2 on the right side), external Iliac (7 on left and 6 on the right side), internal Iliac (1 on the left side) and paravesical (1 on the right side) regions (see Fig. 2). An example of pre-operative imaging findings is shown in Fig. 3.

There was no statistically significant difference (*p* = 0.466) in SN visualization on SPECT/CT between patients who received neoadjuvant therapy (42% non-visualization) and those who did not (33% non-visualization).

### Intra-operative SN identification

A total of 35 SNs were identified during surgery. Of the 31 SNs visualized on SPECT/CT, 30 were successfully excised. Only one SN could not be removed during the open procedure due to tissue adhesions (possibly resulting from neoadjuvant therapy).

During robotic procedures, an additional 4 SNs were identified based on fluorescent signals in 3 patients, located in the para-vesical, external iliac, obturator, and Marcille regions, respectively all 4 nodes were both fluorescent as well as radioactive. In another patient who underwent open cystectomy, ex vivo evaluation of the PLND specimen revealed a radioactive SN.

The PLND yielded a median of 17 LNs per patient, with a total of 592 LNs counted by the pathologist.

### Pathological tumor identification

Following pathological examination, 53% of patients (16/30) presented with a tumor stage of pT2 or higher. A total of 23% of patients (7/30) were classified as pT0, among these, 13% (4/30) achieved ypT0 status, denoting a complete pathological response to NAC. Post-surgical pathological evaluation identified additional concomitant CIS in the bladder in 23% of patients (7/30).

In total 7 tumor positive LNs were recovered from either the SN or PLND specimens. 5 SNs (14%) were found to harbor metastatic disease in 4 patients. Of these, 4 positive SNs were located in the external iliac region, and 1 positive SN was identified in the obturator fossa. Importantly, in these 4 patients, the SN was the only lymph node found to contain metastasis after pathological evaluation.

**Fig. 4:**
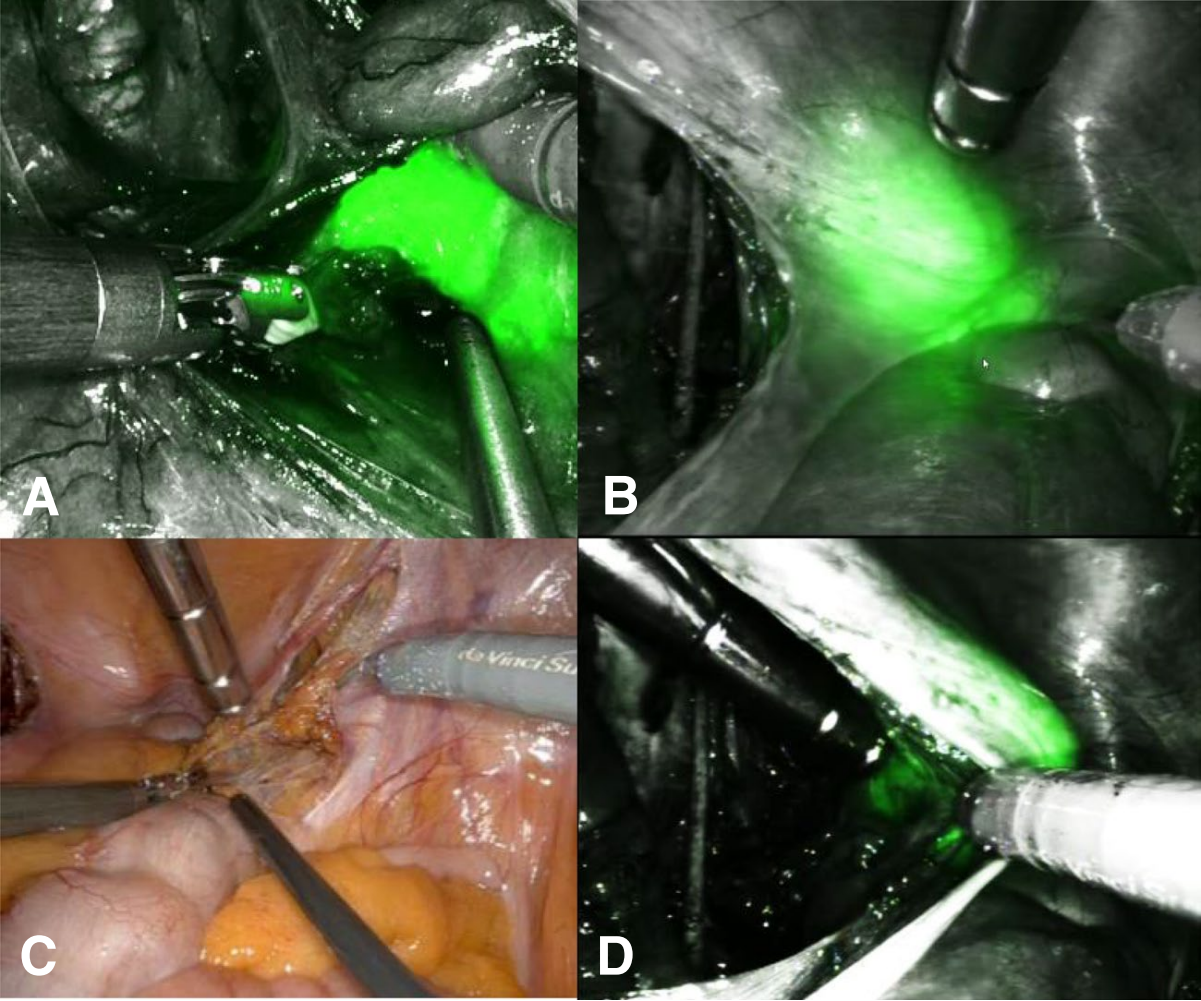
Example of robotic sentinel node identification using a fluorescence camera in combination with a drop-in gamma probe. **A** and **B** Intraoperative fluorescence detection with Firefly fluorescence laparoscope. **C** and **D** intraoperative of image of localizing a sentinel node with C with gamma Drop-in probe and in D same node confirmed with fluorescence imaging

PLND specimens revealed an additional 2 tumor positive LN’s (6% on patient base) that were not visible as SNs on preoperative or intraoperative imaging. In this cohort a total of 4 patients were identified with pN + nodal status. Among these patients, 1 patient did receive NAC with Gemcitabine/Cisplatinum, while 3 patients did not (Fig. 4).

### Survival analysis

**Fig. 5:**
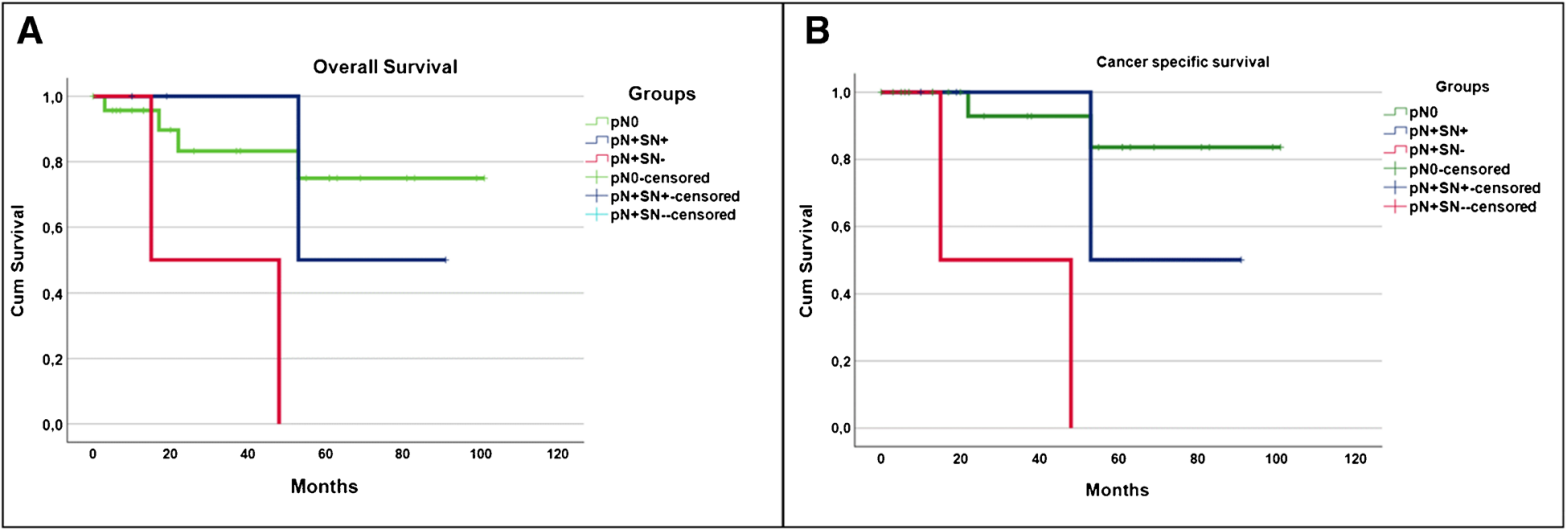
Kaplan Meier survival curves. Kaplan–Meier curves of (**A**) Overall survival (log-rank test, *p* value = 0.015), **B** Cancer specific survival (log-rank test, *p* value < 0.001). Abbreviations: Left overall survival, right cancer specific survival. Red curve = pN + patients with no visualization of SN, Blue curve = pN + with tumor positive SN, Green curve is pN0 patients

Mean follow-up was of 82 months (SD ± 7.1 months). Seven patients (23.3%) died during follow-up, among whom 1 for unknown cause and 1 of complications following cardiac surgery. The remaining 5 patients died of BC-progression. The Kaplan–Meier curves are shown for both overall survival (A) and cancer specific survival (B) in Fig. 5. Survival analyses showed that 2 patients with a non-visualization at SPECT/CT with a positive LN in the PLND (pN + /SN -) died within 5 years after cystectomy. Instead, the 4 pN + patients, in whom the SN was the only positive LN and the complementary PLND showed no LN-metastases presented a much more favorable survival outcome of 75% (pN + /SN +). The pN0- patients had the most favorable survival during total follow up (92%).

The log-rank test for both cancer specific survival and overall survival resulted in a statistically significant difference between the groups (*p* values < 0.001 and 0.015).

## Discussion

This prospective study yielded a total SN detection rate of 73% (63% based on pre-operative imaging and additional 10% based on intraoperative imaging), this was a higher rate compared to our previous report [19]. Although there were no changes to the injection protocol, the current analysis showed increased SN visualization. This might be attributed to a learning curve in performing cystoscopic injections of the hybrid tracer. Another possible explanation is that the last 10 patients underwent robotic surgery, which may have facilitated the detection of additional SN due fluorescence imaging. Despite this improvement, there was still a 37% non-visualization rate at preoperative imaging, a significant part of the current study population, which indicates further procedural optimization is required.

Literature reports visualization rates for SN-identification ranging from 38 to 100%, with sensitivities varying between 50 and 100%. The observed variability may be related to the absence of a universally accepted optimal tracer and techniques for SN-identification in bladder cancer [11, 12, 19, 21, 22, 24]. There is controversy around the use of SN biopsy in bladder cancer because the variation in detection rate and sensitivity [11, 19, 21, 22]. Nevertheless, our results underscore the potential of SN detection to improve nodal staging and guide therapeutic decisions in bladder cancer [11]. For patients with pN + (node-positive SN) findings, the SN was the sole node involved, indicating that in these cases, a PLND and the possible risk on complications could be avoided. Interestingly, patients who had preoperative SN visualization and tested positive for nodal involvement had significantly better survival outcomes (75% vs. 0%) compared to the node-positive patients who did not have preoperative SN visualization. These data support the role of SN biopsy as an accurate reflection of the general nodal status in patients with successful preoperative SN detection [19, 23].

Recently the SWOG S1011 trial showed a local recurrence in 23–35% of patients treated with cystectomy and PLND. A SN could possibly be a more targeted approach compared to a standard PLND to identify the tumor bearing LN in the pelvic region [4].

The controversy surrounding the use of SN biopsy in bladder cancer remains unresolved [11, 12, 19, 21, 22, 24]. Our findings indicate that non-visualization on preoperative imaging is a clear limitation as it still demands more invasive PLND to assess nodal involvement. In this study the chance of missing positive LN in case of non-visualization of SN and not performing a PLND was 2/11 patients (18%). Neoadjuvant systemic therapy was not related to the chance of a possible non-visualization. Although both SN non- visualization patients where in the ePLND a positive LN was found, were treated with NAC. This suggests that NAC may have been associated with a lower incidence of positive SN in this patient population.

Also the current study is limited by its focus on histologically confirmed MIBC or vhNMIBC (cN0M0) and it would make sense to evaluate the accuracy of SN biopsy in patients with smaller tumors (T1/T2). The role of SN in bladder sparing strategies remains unexplored. In prostate cancer, SN biopsy was shown to improve selection of men that migh benefit of pelvic radiotherapy [25].

A central debate in BC and other malignancies is whether patients receiving NAC or neo adjuvant immunotherapy should undergo SN biopsy. The concern is that NAC might impair SN detection and increase the false-negative rate [26]. In the present data NAC did not significantly affect preoperative visualization of SN. An observation that corroborates earlier findings by Rosenblatt et al. [27], where NAC did not affect SN visualization in MIBC. However, it should be noted that both non-visualization patients with a positive LN in the complementary PLND received NAC, and therefore an impact of NAC on SN accuracy can not be excluded.

Based on the tumor characteristics and form of neo-adjuvant therapy, this is a heterogeneous group. Although in this study a small and heterogeneous group was analyzed, the survival curves (Fig. 5) based on pN status show the importance of nodal staging on the prognosis in BC patients. This highlights the relevance of nodal staging and the potential for a future optimized SN procedures in BC patients.

## Conclusion

This prospective study demonstrated that hybrid SN procedures in cN0M0 bladder cancer patients could reliably predicted the overall nodal status in the majority of cases. For patients with positive SN, a complementary PLND did not reveal additional nodal metastase and could have been avoided. Conversely, in patients with non-visualization, PLND still provided value. Outcome of patients with nodal metastases detected by SN was improved compared to patient with nodal metastases and non-visualization of SN. Further research is warranted to improve the SN visualization rates and assess its applicability in earlier-stage tumors (T1/T2) and bladder-sparing approaches.

## Data Availability

The datasets generated during and/or analysed during the current study are available from the corresponding author on reasonable request.
